# Supplementary material for: Identification of photoperiod-induced specific miRNAs in the adrenal glands of Sunite sheep (Ovis aries)
Source: Front Vet Sci. 2022 Jul 22;9:888207. doi: 10.3389/fvets.2022.888207 (PMC9354845; doi:10.3389/fvets.2022.888207)
Supplement: Supplementary file 1 [file Data_Sheet_1.ZIP › Supplementary file/miRDeep2 file.zip!/miRDeep2 file/Know_Structure/LP42A1/oar-mir-29b-1.pdf]

miRBase precursor : oar-mir-29b-1
Total read count : 12024
oar-miR-29b read count : 11080
remaining reads : 944

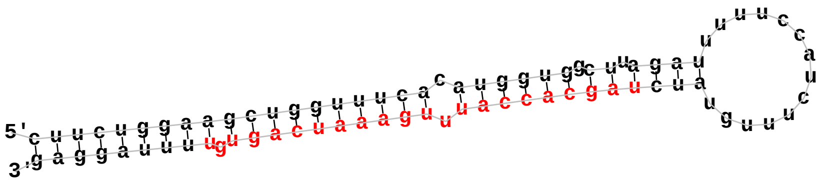

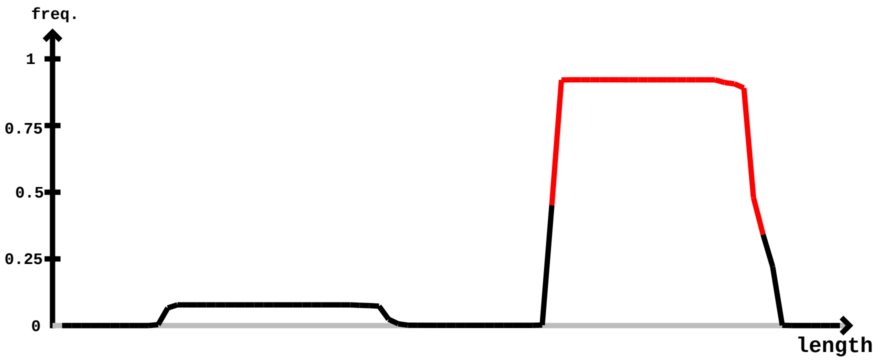

oar-miR-29b

```
5'- cuucuggaagcugguuucacaugguggcuuagauuuuuccaucuuuguaucuagcaccauuugaaaucaguguuuuaggag   -3'    exp
    ((((((((((((((((((.((((((.((.((((................)))))))))))).)))))))))).))))))))    reads      mm         sample
    .........gcugguuucacaugguggcuu...................................................    2          0          seq
    .........gcugguuucacaugguggcuua..................................................    2          0          seq
    .........gcugguuucacaugguggcuuag.................................................    4          0          seq
    .........gcugguuucacaugguggcuuaga................................................    34         0          seq
    ..........cugguuucacaugguggcuu...................................................    16         0          seq
    ..........cugguuucacaugguggcuua..................................................    13         0          seq
    ..........cugguuucacaugguggcuuag.................................................    16         0          seq
    ..........cugguuucacaugguggcuuaga................................................    496        0          seq
    ..........cugguuucacaugguggcuuagau...............................................    159        0          seq
    ..........cugguuucacaugguggcuuagauu..............................................    49         0          seq
    ..........cugguuucacaugguggcuuagauuu.............................................    3          0          seq
    ..........cugguuucacaugguggcuuagauuuu............................................    1          0          seq
    ...........ugguuucacaugguggcu....................................................    1          0          seq
    ...........ugguuucacaugguggcuuag.................................................    4          0          seq
    ...........ugguuucacaugguggcuuaga................................................    72         0          seq
    ...........ugguuucacaugguggcuuagau...............................................    45         0          seq
    ...........ugguuucacaugguggcuuagauu..............................................    13         0          seq
    ...........ugguuucacaugguggcuuagauuu.............................................    2          0          seq
    ...........ugguuucacaugguggcuuagauuuuu...........................................    1          0          seq
    .................................uuuuuccaucuuuguauc..............................    10         0          seq
    ....................................uuccaucuuuguaucuagcacc.......................    1          0          seq
    .................................................ucuagcaccauuugaaauc.............    1          0          seq
    .................................................ucuagcaccauuugaaaucGg...........    1          1          seq
    .................................................ucuagcaccauuugaaaucagu..........    10         0          seq
    ..................................................cuagcaccauuugaaau..............    2          0          seq
    ..................................................cuagcaccauuugaaauc.............    55         0          seq
    ..................................................cuagcaccauuugaaauca............    14         0          seq
    ..................................................cuagcaccauuugaaaucGg...........    1          1          seq
    ..................................................cuagcaccauuugaaaucag...........    22         0          seq
    ..................................................cuagcaccauuugaaaucagu..........    2921       0          seq
    ..................................................cuagcaccauuugaaaucGgu..........    31         1          seq
    ..................................................cuagcaccauuugaaaucagug.........    1286       0          seq
    ..................................................cuagcaccauuugaaaucagugu........    1069       0          seq
    ..................................................cuagcaccauuugaaaucaguguu.......    6          0          seq
    ...................................................uGgcaccauuugaaauc.............    1          1          seq
```

oar-miR-29b

```
cuucuggaagcugguuucacaugguggcuuagauuuuuccaucuuuguaucuagcaccauuugaaaucaguguuuuaggag
...................................................uagcaccauuugaaauU.............   6      1      seq
...................................................uagcaccauuugaaauA.............   1      1      seq
...................................................uagcaccauuugaaauc.............   58     0      seq
...................................................uagcaccauuugaaaucG............   29     1      seq
...................................................uagcaccauuugaaauca............   14     0      seq
...................................................uagcaccauuugaaaucag...........   44     0      seq
...................................................uagcaccauuugaaaucGg...........   115    1      seq
...................................................uagcaccauuugaaaucGgu..........   649    1      seq
...................................................uagcaccauuugaaaucagu..........   1333   0      seq
...................................................uagcaccauuugaaaucagug.........   379    0      seq
...................................................uagcaccauuugaaaucagugu........   384    0      seq
...................................................uagcaccauuugaaaucaguguu.......   2628   0      seq
...................................................uagcaccauuugaaaucaguguuu......   10     0      seq
...................................................uagcaccauuugaaaucaguguuC......   1      1      seq
...................................................uagcaccauuugaaaucaguguuuu.....   1      0      seq
....................................................agcaccauuugaaaucagu..........   2      0      seq
....................................................agcaccauuugaaaucGgu..........   1      1      seq
....................................................agcaccauuugaaaucaguguu.......   5      0      seq
```
